# Electron beam radiotherapy for the management of squamous cell carcinoma of the anal margin

**DOI:** 10.2340/1651-226X.2024.40199

**Published:** 2024-08-08

**Authors:** Lars Fokdal, Bjarke Mortensen, Lars Henrik Jensen, Mette Møller Sørensen, Sean Patrick McIlroy, Birgitte Mayland Havelund

**Affiliations:** a Department of Oncology, Vejle Hospital, University Hospital of Southern Denmark, Denmark; b Department of Regional Health Research, University of Southern Denmark, Odense, Denmark; c Radiotherapy Research Team, Department of Oncology, Vejle Hospital, University Hospital of Southern Denmark, Denmark; d Department of Surgery, Vejle Hospital, University Hospital of Southern Denmark, Denmark

**Keywords:** Squamous cell carcinoma of the anal margin, anal cancer, radiotherapy, electron beam radiotherapy

## Abstract

**Purpose and objective:**

Squamous cell carcinoma of the anal margin (SCCAM) is an uncommon lesion that comprises one-third to a quarter of all anal squamous cell carcinoma. Treatment involves surgery or exclusive radiotherapy for small tumours, whereas the preferred treatment for larger tumours is chemoradiotherapy. In our department, selected patients with SCCAM are treated with electron beam radiotherapy using one perineal field. The present study evaluates this strategy.

**Material and methods:**

All consecutive patients with SCCAM and treated with electron beam radiotherapy from 2012 to 2022 were included. Data were retrospectively extracted from the medical records and analysed descriptively. Local control (LC) and overall survival (OS) were analysed using Kaplan-Meier statistics.

**Results:**

Forty patients were evaluated. Primary radiotherapy was delivered in 35 (87.5%) patients. Five (12.5%) patients had postoperative radiotherapy. Median prescription dose was 60.0 (range 45.0–60.2) Gy in 28 (range 10–30) fractions delivered with 8 (range 4–18) MeV using a standard circular aperture and bolus.

At a median follow-up of 73 (range 9–135) months, 7 (17.5%) patients were diagnosed with local recurrences. The 5-year LC rate was 84.3% (95% CI: 71.4%–97.2%). Analysis of LC according to T-stage revealed a 5-year LC of 100% (95% CI: 100%–100%) in T1 tumours compared to 57.0% (95% CI: 27.4%–86.6%) in T2 tumours (*p* < 0.001). 5-year OS was 91.6% (95% CI: 83.0%–100%). Late grade 3 toxicity included ulceration in the skin and subcutis in 2 (5.0%) patients.

**Intepretation:**

Electron beam radiotherapy enables the delivery of ‘eye-guided’ radiotherapy directly to the tumour. LC is good in patients with T1 tumours. Patients with T2 tumours have less satisfactory LC and should be treated with chemoradiotherapy.

## Introduction

Squamous cell carcinoma of the anal margin (SCCAM) is an uncommon lesion that comprises one-third to a quarter of all anal squamous cell carcinomas (SCCAs) [1].

Chemoradiotherapy is the current standard of care in a majority of all cases [2–4]. In selected patients with T1 tumours, radiotherapy alone, or surgery with a local excision are possible treatment options [5–7]. The choice of treatment modality should be based on the probability of disease control versus the risk for toxicity, including the anticipated function of anal sphincter [1, 3, 8, 9].

In our department, all patients with SCCAM are jointly evaluated by a clinical oncologist and a colorectal surgeon. Patients who present with a T1 tumour at an adequate distance from the anal verge to perform a local excision are selected for surgery. All other patients are referred for (chemo)radiotherapy.

During the last decade, selected patients with SCCAM who present with T1–T2 lesions close to the anal verge have been treated with electron beam radiotherapy with a single perineal field. This technique offers the opportunity to plan and deliver ‘eye-guided’ radiotherapy directly to the perianal tumour without posing technical challenges. This is an advantage, especially in small SCCAM where the lesion may be difficult to define during contouring and 3-dimensional (3D) dose planning not only on computer tomography (CT) scan, but also on positron emission CT (PET-CT) and magnetic resonance imaging (MRI).

Electron beam radiotherapy utilising a single perineal field minimises the risk of geographical miss during the planning and delivery of treatment but does not deliver prophylactic radiotherapy to the elective lymph nodes. This is concerning as the regional lymph nodes remain untreated for potential micrometastases, elevating the risk of regional recurrence.

The present retrospective study was initiated to investigate the role of electron beam radiotherapy with a single perineal field in patients with SCCAM. The general aim was to investigate treatment outcomes in terms of locoregional control, overall survival (OS), and morbidity in patients who received treatment within the last decade.

## Material and methods

### Patients for the study

In total, 320 consecutive patients with SCCA were referred for treatment at the Department of Oncology, Vejle Hospital, Denmark, between October 2012 and October 2022. Vejle Hospital is one of three specialised centres in Denmark dedicated to the treatment of this rare disease. All patients underwent a staging procedure comprising PET-CT and MRI scans, and an ultrasound (US) examination of the groins. Biopsies were conducted in case of suspicious lymph nodes. In addition, a clinical examination with biopsy of the primary tumour was performed in general anaesthesia with the participation of a colorectal surgeon and a clinical oncologist. Anoscopy was done in patients where involvement of the anal canal was suspected.

After initial staging, 55 (17.2%) of all patients were selected for palliative treatment due to advanced stage disease or medical comorbidity. The remaining 265 (82.8%) patients were offered (chemo)radiotherapy. A total of 40 (15.1%) of these patients were selected for electron beam radiotherapy. The selection criteria included T1–T2 lymph node negative tumours that were located in the perianal skin and completely visible on clinical examination. The remaining 225 (84.9%) patients were treated with (chemo)radiotherapy to the tumour and elective lymph node regions.

### Electron beam radiotherapy

Radiotherapy was planned and delivered with the patient in the lithotomy position. The gross target volume (GTV) for radiotherapy included all visible and palpable tumour. The clinical target volume (CTV) was defined as the GTV with a 5 mm isotropic margin. In addition, a 5 mm isotropic margin was added from the CTV to the planning target volume (PTV), and a 10 mm isotropic margin was added from the PTV to the edge of the aperture to ensure coverage of the PTV with at least 85%–90% of the prescribed dose.

Radiotherapy planning and delivery was ‘eye-guided’ and included a single electron field using a standard circular aperture and a 3–5 mm bolus. Dose and fractionation schedule changed during the inclusion period of patients for the study (Table 2).

**Figure 1:**
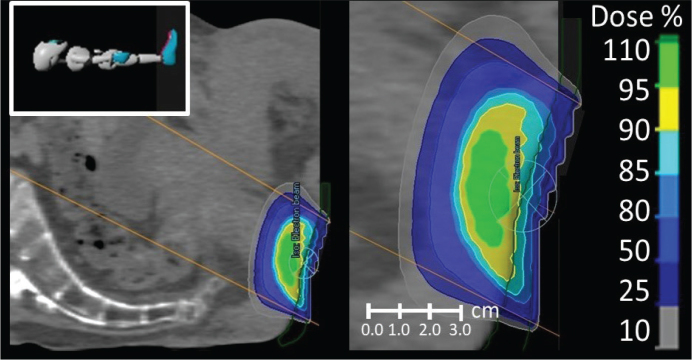
Electron beam radiotherapy using one perineal field for the treatment of squamous cell carcinoma of the anal margin. The figure has been made for illustrative purposes and shows isodose lines for 60.2 Gy in 28 fractions with standard circular 60 mm aperture with 8 MeV electron beam and 5 mm bolus in green.

The prescription of electron energy was based on the thickness of the tumour to ensure coverage (Figure 1). Dose calculation was done by a simple factor-based monitor unit calculation. No 3D dose planning was done.

### Post-treatment follow-up

Follow-up after radiotherapy was carried out every 3 months in the first year, every 4 months the second year followed by assessments every 6 months for the subsequent 2 years, and then annually until a total of 5 years. Treatment response evaluations included PET-CT and MRI, at 3-month follow-up, followed by annual assessments thereafter.

### Data collection and variables

Clinically relevant patient-, disease-, and treatment-related data and morbidity were extracted from medical records. Moreover, disease outcomes (local control [LC], regional control, systemic control), OS, and salvage treatment were recorded.

### Statistical analysis

Disease outcomes were analysed with Kaplan Meier statistics and log-rank test. Follow-up was calculated as the time from diagnosis until an event (recurrence or death) or last follow-up, whichever came first. Patients were censored at the last follow-up or at the time of an event. Morbidity was scored according to the Common Terminology Criteria for Adverse Events version 4.0 (CTCAE, V4.0) and analysed in crude numbers. The SPSS statistical software system v.20 (IBM SPSS Statistics for Windows, Version 20.0 Armonk; NY: IBM Corp.) was used for statistical analysis.

## Results

Baseline characteristics for the 40 patients are listed in Table 1. Briefly, 10 males (25.0%) and 30 females (75.0%) were included. Median age was 66 (37–84) years, and 70.0% had comorbidity, but all patients had performance status of 0–1. The distribution of T-stage was 65.0% and 35.0% for T1 and T2 tumours, respectively.

**Table 1:** Patient and disease characteristics. Tumour dimensions were assessed during clinical examination while lymph node stage was assessed with imaging.

Patient and disease characteristics		*N* = 40
Median age, years (range)		66 (37–84)
Gender (%)	Female	30 (75.0)
	Male	10 (25.0)
Tobacco (%)	Smoker	19 (47.5)
	Previous smoker	9 (22.5)
	Non-smoker	12 (30.0)
Charlson comorbidity index (%)	None	12 (30.0)
	Mild	20 (50.0)
	Moderate	7 (17.5)
	Severe	1 (2.5)
ECOG performance status (%)	0	27 (67.5)
	1	13 (32.5)
	2–4	0 (0)
Prior tumour resection (%)	No	35 (87.5)
	Yes (R1 resection)	5 (12.5)
Tumour stage (%)	1 (≤ 20 mm.)	26 (65.0)
	2 (20–50 mm.)	14 (35.0)
Tumour diameter mm. (range)		19 (5–30)
Tumour thickness mm. (range)		5 (3–25)
Tumour involvement of the anal canal (%)	No	29 (72.5)
	Yes	11 (27.5)
P16-positive (%)	Yes	24 (60.0)
	No	3 (7.5)
	Unknown	13 (32.5)
Lymph node stage (%)	0	40 (100)
	1	0 (0.0)

Five patients (12.5%) had postoperative radiotherapy after an R1 resection for a T1 tumour, and the remaining 35 patients (87.5%) had primary radiotherapy. Electron beam radiotherapy was delivered with standard tubes with a median diameter of 60 mm (range 40–90) and 8 MeV (range 4–18). The radiotherapy dose was 60.0 Gy (range 45.0–60.2) delivered in 28 fractions (range 10–30). Detailed information regarding radiotherapy is available in Table 2.

**Table 2:** Radiotherapy-related parameters.

Dose and fractionation (EQD_2Gy_, α/β = 10 Gy)	Number of patients (%)	Tumour stage (#patients)	Median tumour size (range) [mm]	Local failure	Median aperture (range) [mm]	Median Energy (range) [MeV]
50 Gy/25 fx (50.0 Gy)	6 (15.0)	T1: 6	7.5 (5–10)	0	45 (40–60)	6 (4–8)
		T2: 0		0		
45 Gy/10 fx (54.4 Gy)	1 (2.5)	T1: 1	NA	0	60 (NA)	6 (NA)
		T2: 0		0		
53.75 Gy/25 fx (54.4 Gy)	5 (12.5)	T1: 4	15 (8–30)	0	50 (40–90)	6 (6–8)
		T2: 1		0		
56 Gy/28 fx (56 Gy)	2 (5.0)	T1: 1	15 (10–20)	0	55 (50–60)	9 (8–10)
		T2: 1		1		
60 Gy/30 fx (60 Gy)	7 (17.5)	T1: 6	15 (8–30)	1	80 (70–90)	12 (6–18)
		T2: 1		0		
60.2 Gy/28 fx (61.0 Gy)	19 (47.5)	T1: 8	21 (10–30)	1	60 (40–80)	8 (6–15)
		T2: 11		4		
Total number of patients	40 (100)	T1: 26	18.5 (5–30)	2	60 (40–90)	8 (4–18)
		T2: 14		5		

The 3 month follow-up examination showed a complete response for all patients. At a median follow-up of 73 months (range: 9–135), 7 patients (17.5%) had been diagnosed with local recurrences; three patients with T2 tumours had local recurrence only; two patients with T2 tumours had local and simultaneous groin recurrence; two patients with T1 tumours had local recurrence only diagnosed after 64 months and 113 months, respectively. No isolated regional or metastatic recurrences were diagnosed.

**Figure 2:**
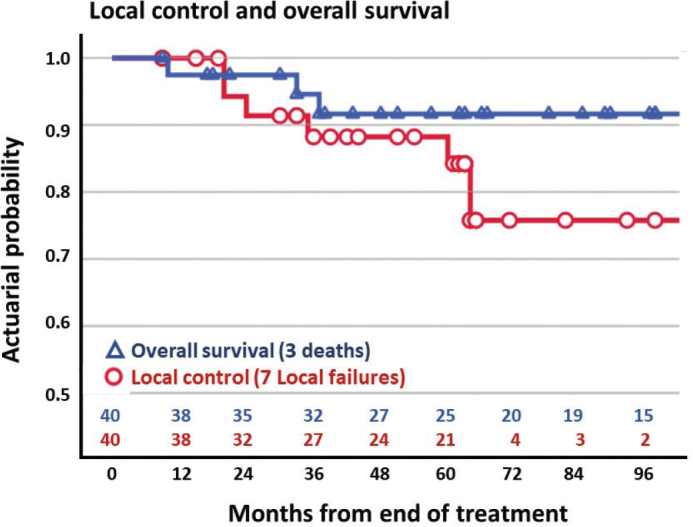
Overall survival (blue) and local control (red) in 40 patients treated with electron beam radiotherapy for squamous cell carcinoma of the anal margin.

**Figure 3:**
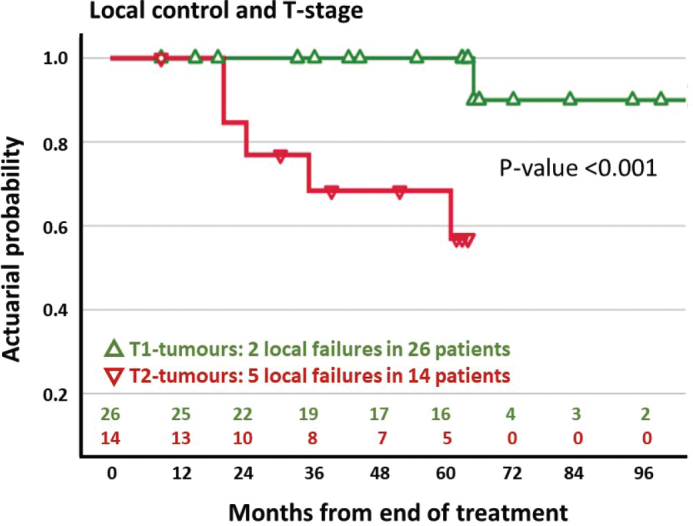
Local control and tumour stage in 40 patients treated with electron beam radiotherapy for squamous cell carcinoma of the anal margin. Patients are classified in T1-tumours (green) and T2-tumours (red).

The 5-year LC was 84.3% (95% CI: 71.4%–97.2%) (Figure 2). Analysis of LC according to T-stage revealed a 5-year LC of 100% (95% CI: 100%–100%) in T1 tumours compared to 57.0% (95% CI: 27.4%–86.6%) in T2 tumours (Figure 3).

Salvage surgery including vertical rectus abdominis musculocutaneous flap (VRAM) reconstruction was offered to 6 patients with local recurrence. One patient was offered re-irradiation. After salvage treatment, one patient developed liver metastases. The remaining patients did not experience any later recurrence (Table 3).

**Table 3:** Outcome of salvage treatments in 7 patients with local (n = 5) and locoregional (n = 2) recurrent SCCAM.

Patient ID	Time to failure (months)	Type recurrence	P16-positive	T-stage	Involvement Anal canal	RT Dose (Gy) /F	Salvage treatment	Later recurrence
11	20	Local	**+**	2	**+**	60.2/28	Surgery	No
19	60	Local	**+**	2	**+**	56.0/28	Surgery	No
25	64	Local	**+**	1	**+**	60.2/28	Surgery	No
26	24	Local + regional	**+**	2	**-**	60.2/28	Surgery	Liver metastases
31	35	Local + regional	**+**	2	**-**	60.2/28	Surgery	No
37	20	Local	**-**	2	-	60.2/28	Surgery	No
53	113	Local	**+**	1	-	60.0/30	Reirradiation	No

Colostomy-free survival at 5 years was 83.0% (95% CI: 70.5%–95.5%) in all patients. In the 33 patients without local recurrence, the 5-year colostomy-free survival was 93.7% (95% CI: 85.3%–100%).

On univariate analysis, the T-stage was associated with LC (*p* < 0.001). No other patient-related (gender, tobacco, performance status, tumour thickness/diameter) or treatment-related factors (dose, fractionation, tube aperture diameter, electron beam energy) were associated with LC.

During follow-up, three patients died: two patients due to anal cancer, and one patient due to other causes. The 5-year OS was 91.6% (95% CI 83.0%–100%) (Figure 2).

Acute morbidity included grade 1–2 pain and moist desquamation in the skin in 23 patients (57.5%). All patients completed treatment as planned without interruptions.

During follow-up, two patients (5.0%) developed grade 3 toxicity with ulceration in the skin and subcutaneous tissue. Both patients had surgery with a stoma. Late grade 2 toxicity was reported in three patients (7.5%): two patients with anal incontinence and one patient with anal stenosis. The two patients with grade 3 ulceration and one patient with grade 2 incontinence were treated with high electron beam energy in the range of 15–18 MeV in contrast to the remaining patients who were treated with electron energy in the range of 6–12 MeV.

## Discussion

Squamous cell carcinoma of the anal margin is a rare malignancy that calls for centralisation and treatment within a multidisciplinary team [1]. The choice of treatment depends on several factors including the location and stage of the tumour, the anticipated functional results, and the risk of complications. Surgery with a wide local resection is a treatment option for small tumours that do not involve the anal canal [1, 5, 7, 10, 11]. For all other patients, the preferred treatment includes chemoradiotherapy in larger tumours while small tumours may be treated with radiotherapy alone [1–4, 6, 8, 12–15].

Our study as well as previous investigations in SCCAM are limited by a retrospective design with inclusion of small numbers of patients who have been treated with different (chemo)radiotherapy schedules and techniques. Due to these limitations, it is difficult to compare studies directly and draw firm conclusions regarding the optimal treatment.

The present study confirms that electron beam radiotherapy is a good treatment option in selected patients with SCCAM. In the whole group of patients, the 5-year LC rate was 84.3%, while the OS was 91.6%. These results are in line with previous studies that investigate outcomes of radiotherapy in SCCAM. In these studies, LC rates range from 61% to 88%, while OS rates of 55% to 82% were found at 5-year [2, 8, 12–15].

Analysis of LC in accordance with T-stage showed an excellent 5-year LC rate of 100% in patients with T1 tumours. Two local recurrences were diagnosed after more than 5-years follow-up (Table 3). A majority of all SCCA recurrences are diagnosed within the first 3-years follow-up [16] and it could be argued that the two patients had a second primary tumour rather than a recurrence.

Patients with T2 tumours had a significantly lower LC rate of 57% at 5 years compared to patients with T1 tumours. This is expected because T-stage is a prognostic factor for LC [12]. The LC rate in patients with T2 tumours in the present study is unsatisfactory compared to previous studies that include patients with advanced stage disease with a less favourable prognosis [2, 8, 12–15].

The management of inguinal lymph nodes in SCCAM is controversial. Lymph node involvement is a known prognostic factor [1, 8, 12]. In some centres, prophylactic lymph node irradiation is performed in all patients [10, 13], while other centres do not [12, 15]. Electron beam radiotherapy with one perineal field enables ‘eye-guided’ treatment but does not sterilise potential lymph node metastases. This is a concern, because the potential benefit of ‘eye-guided’ radiotherapy may be outweighed by an increased risk for a regional recurrence.

The risk for lymph node involvement is related to the size of the primary tumour. In a previous study, the distribution of lymph node metastases was investigated in 57 patients with SCCAM. Inguinal lymph node metastases were found in 0% (0 events/10 patients) of patients with T1 tumours, 24% (9 events /38 patients) of patients with T2-tumours, and 67% (6 events /9 patients) of patients with T3–T4 tumours. Based on these results, the authors recommended elective irradiation of the inguinal lymph nodes in patients with lymph node negative tumours, except for T1 lesions [2]. In another retrospective study, investigating 32 patients with anal margin cancer, inguinal lymph node irradiation was recommended in lymph node negative patients if the primary tumour was larger than 4 cm [15].

Based on existing literature, a recent review investigated radiotherapy treatment volumes in early stage (T1–T2 N0 M0) SCCAM. They recommended that the delineation of the primary should be based on a clinical assessment including anorectal endoscopy and imaging with MRI and PET-CT. With respect to elective treatment volumes, the ano-inguinal region should be considered on a case-by-case basis, while elective lymph node irradiation is generally recommended, except for well-differentiated T1 tumours where it may be omitted [17].

In our study, two patients with T2 tumours were diagnosed with a local and simultaneous groin recurrence in contrast to patients with T1 tumours where no lymph node recurrences were found.

Based on these results, and the results from previous studies as well as guidelines [2, 6, 7, 15, 17], we suggest that electron beam radiotherapy with a perineal field alone is a durable treatment option in patients diagnosed with a T1 N0 M0 tumour after a thorough clinical assessment and imaging with MRI and PET-CT. This is in contrast to patients with T2 tumours who have a considerable risk for lymph node metastases and local recurrence [2, 17]. These patients should be treated with chemoradiotherapy with elective lymph node irradiation [18–20].

In the present study, a majority of all local recurrences were managed with salvage surgery including VRAM reconstruction. One patient with a local and simultaneous groin recurrence died of systemic disease after salvage surgery; the remaining patients did not experience any new recurrences. This is in agreement with other studies that investigated the outcome of salvage surgery. In these studies, acceptable cure rates for patients with solitary local recurrences were found in contrast to patients with a local and simultaneous regional recurrence [2, 8].

Late morbidity in our study included grade 3 ulceration or grade 2 anal stenosis or incontinence in two (5.0%) and three (7.5%) patients, respectively. Evaluation of the electron beam energy prescriptions showed that three patients (7.5%) were treated with high electron energies in the range from 15 to 18 MeV to include the whole anal canal within the prescription isodose. Two of these patients developed grade 3 ulceration, while the third patient developed grade 2 incontinence. All other patients were treated with lower electron beam energies in the range from 6 to 12 MeV. Based on these results, the use of high electron beam energy is not recommended in the treatment of SCCAM. For patients where the whole anal canal is considered the target, treatment should include photon beam therapy with 3D dose planning.

Previous studies on SCCAM have reported late morbidity in line with our results. In one study, 45 patients were evaluated after external beam radiotherapy (EBRT) alone (*n* = 36) and/or brachytherapy (*n* = 9). The results of the study showed grade 3–4 perineal ulceration in 5.0% of all patients, while grade 3 anal stenosis and anal incontinence were found in 5.0% and 7.0% of the patients, respectively [8].

In another study, two (8.0%) out of 24 patients had grade 3–4 ulceration necessitating surgery. Both patients had been treated with a brachytherapy boost [14].

In a third study, which investigated 26 patients, where treatment included EBRT alone (*n* = 21) and/or brachytherapy (*n* = 5), 6 patients (23.0%) experienced grade 3–4 late morbidity with necrosis and/or incontinence. It was concluded that the high incidence of severe morbidity was attributed to the use of a hypo-fractionated radiotherapy schedule [12].

In our study, 5-year colostomy-free survival was 93.7% in patients without recurrence. Previous studies have found similar results and report sphincter preservation rates ranging from 66% to 92% in cured patients [2, 4, 8, 12, 15], which illustrate that radiotherapy is associated with good functional outcome in most patients.

In the present study, patients were treated without concomitant chemotherapy. This is in contrast to most previous studies, where a proportion of all patients were treated with chemoradiotherapy. In these studies, it has not been possible to draw valid conclusions regarding the effects of concomitant chemotherapy, because patients who were selected for chemoradiotherapy were more likely to have advanced stage disease [2, 3, 8, 10, 12–15].

In locally advanced SCCA, two prospective randomised trials have demonstrated a survival benefit of concomitant chemotherapy to radiotherapy [18–20]. Although there are no specific prospective data that investigate the role of concomitant chemotherapy in SCCAM, it is important to notice that SCCAM constituted 23% of the patient population in one of the two studies [20]. In the other study, no difference in LC and OS was found between the anal margin and anal SCC. Therefore, the results from both studies have been considered applicable to all SCCA including SCCAM. This is also in agreement with clinical practice in the two latest series of patients with advanced stage SCCAM where concomitant chemotherapy is considered as standard and routinely used [3, 4].

In our study as well as previous studies of patients with SCCAM, different dose- and fractionation schedules have been used [8]. The heterogeneity in dose prescription reflects the fact that patients have been included over a considerable time span during which radiotherapy schedules have changed. Moreover, in many studies, radiotherapy has been prescribed based on a risk-adapted approach with lower prescription doses in small tumours compared to larger tumours (Table 2).

In our study, two patients with T1 tumours had a local recurrence after a 5-years follow-up. Both patients had a radiotherapy dose ≥60 Gy (EQD2), while all other patients with T1 tumours had doses <56 Gy (EQD2). If these two recurrences were classified as second primaries rather than primary recurrences, it would imply that a radiotherapy dose <56 Gy (EQD2) was enough to cure patients with T1 tumours. This is contrary to patients with T2 tumours, where 4 out of 5 local recurrences were diagnosed in patients treated at 61 Gy (EQD2) which suggests that these patients should receive a more intensive treatment than electron beam radiotherapy.

In a previous study, no evidence for improved LC was found in 45 patients with SCCAM when the dose exceeded 59.4 Gy. However, the risk of complications increased for doses beyond 59.4 Gy, leading the authors to recommend doses in the range from 55 to 59.4 Gy independently of T-stage [8]. These doses are also in agreement with the prescribed doses in our study. To improve LC in patients with T2 tumours, treatment intensification could include dose-escalation to the primary, but this strategy would increase the risk for severe complications. For future patients with T2 SCCAM, we therefore suggest chemoradiotherapy with photons and prophylactic treatment to the elective lymph nodes.

## Conclusion

Electron beam radiotherapy enables the delivery of ‘eye-guided’ radiotherapy directly to the tumour in patients with SCCAM. The outcome is good in patients with T1 tumours where a high LC rate and acceptable toxicity were found. For patients with T2 tumours, LC is less satisfactory and regional lymph node recurrences are of concern. These patients should be offered chemoradiotherapy with elective lymph node irradiation.

## Data Availability

The authors confirm that the data supporting the findings of this study are available within the article. Raw data that support the findings of this study are available from the corresponding author, upon reasonable request.
